# *Aloe vera* Gel as an Adjunct to Root Surface Debridement in Type II Diabetes With Periodontitis

**DOI:** 10.1155/ijod/5544638

**Published:** 2026-04-15

**Authors:** Chenar A. Mohammad, Hozan W. Aziz, Aveen A. Jalal, Faraedon M. Zardawi

**Affiliations:** ^1^ Periodontics Department, College of Dentistry, Hawler Medical University, Erbil, 44001, Iraq, hmu.edu.iq; ^2^ Faculty of Dentistry, Qaiwan International University, Sulaymaniyah, 00964, Iraq, uniq.edu.iq

**Keywords:** *Aloe vera* gel, chronic periodontitis, diabetes, PGE2, pro-inflammatory cytokines

## Abstract

**Background:**

This study aimed to monitor the effects of subgingival application of *Aloe vera* (ALV) gel on clinical periodontal parameters, pro‐inflammatory cytokines, and the biochemical mediator of inflammation, prostaglandin E2 (PGE2), in patients with type II diabetes and chronic periodontitis.

**Methods:**

Eighty participants with stages 2 or 3 (moderate to severe) periodontitis were included, comprising individuals with partially controlled and uncontrolled (HbA1c >7%) type II diabetes as well as nondiabetic subjects, were randomly divided into four groups (20 in each group) by using the sealed opaque envelope method. They received different treatment modalities: root surface debridement (RSD) combined with either ALV gel or chlorhexidine (CHX) gel. Periodontal parameters and blood samples were obtained at baseline and after 3 months to estimate levels of glycated hemoglobin (HbA1c), PGE2, interleukin‐1 beta (IL‐1β), tumor necrosis factor alpha (TNF‐α), and IL‐6.

**Results:**

ALV gel demonstrated a significant effect on the improvement of clinical periodontal parameters, including plaque index (PI), bleeding on probing (BOP), probing pocket depth (PPD), and clinical attachment level. It also significantly reduced levels of PGE2, fasting blood sugar (FBS), HbA1c, and pro‐inflammatory cytokines after 3 months of therapy in both diabetic and nondiabetic periodontitis patients (*p* < 0.05).

**Conclusions:**

ALV gel exhibits a similar effect to CHX in reducing and improving clinical periodontal parameters, blood sugar levels, PGE2, and cytokine levels. Therefore, ALV gel can be administered locally as an adjunct to RSD in diabetic patients with chronic periodontitis.

## 1. Introduction

Periodontitis and diabetes mellitus (DM) are two prevalent inflammatory diseases affecting people globally. DM is a metabolic disease characterized by prolonged hyperglycemia resulting from deficiencies in insulin secretion or function, or both. Plasma glucose levels for the diagnosis of diabetes are typically estimated using three methods: glycated hemoglobin (HbA1c), an oral glucose tolerance test, or fasting plasma glucose level [1].

Periodontitis is a common oral cavity infection and a major cause of tooth loss in adults. It is considered the sixth most common complication of DM. There is a bidirectional relationship between diabetes and periodontitis, suggesting that systemic disease increases the risk of oral infection and vice versa. Elevated glucose concentrations in diabetic tissues lead to the accumulation of advanced glycation end products (AGEs) through the nonenzymatic glycation and oxidation of proteins, including collagen. These AGEs contribute significantly to the destruction of periodontal tissue [2]. Tumor necrosis factor alpha (TNF‐α) and interleukin‐1 beta (IL‐1β) have been implicated in the pathogenesis of periodontitis. An increased level of TNF‐α is produced when monocytes are stimulated during periodontal inflammation. This cytokine interacts with insulin function and sensitivity through direct and indirect mechanisms, exacerbating the diabetic state and potentially worsening periodontal tissue destruction [3]. The majority of tissue death in periodontitis is caused by the overactivity of TNF‐α and IL‐1β, which are excessively released when the host exhibits an exaggerated immune response against periodontal pathogens [4].

The two main approaches to treating periodontal disease are surgical and nonsurgical. Both involve mechanically removing dental biofilm and calcified deposits, known as supra and subgingival calculus, from the affected root surfaces through scaling and root planing (SRP). However, mechanical debridement may not be efficiently accomplished in cases of furcation involvements and deep pockets due to access limitations. Certain pathogens, such as *P. gingivalis*, *A. actinomycetemcomitans*, B. forsythus, and *P. intermedia*, are difficult to eradicate due to restricted access to deep subgingival areas, which increases the likelihood of treatment failure and disease progression [5].

Antimicrobial agents have been employed as adjunctive therapies to root surface debridement (RSD) in the management of periodontitis for several decades, aiming to enhance clinical outcomes by reducing pathogenic bacterial load [6]. Despite their demonstrated efficacy, the systemic administration of these agents is often associated with adverse side effects, including gastrointestinal disturbances, allergic reactions, and the development of antimicrobial resistance [7]. Consequently, there has been a progressive shift toward localized antimicrobial delivery systems, which facilitate targeted application directly to periodontal pockets, thereby minimizing systemic exposure and associated risks [8].

In recent years, the exploration of natural herbal products as alternative antimicrobial agents has gained considerable interest. These phytochemicals, derived from various medicinal plants, possess antimicrobial, anti‐inflammatory, and regenerative properties, making them promising adjuncts in periodontal therapy [9]. Their use aims to circumvent the limitations of conventional antibiotics, such as resistance development and systemic toxicity, while harnessing the therapeutic potential of bioactive plant compounds. Overall, the evolving landscape of periodontal antimicrobial therapy emphasizes localized delivery and natural alternatives to optimize treatment efficacy and safety.

Numerous alternative therapies and products are considered beneficial, including topical hyaluronic acid application [10]. Additionally, Ayurvedic and herbal medicines have recently drawn particular attention as safe medications with few to no side effects. Because of these advantages, herbal medicine has become popular as a localized medication delivery method for the treatment of periodontitis. *Aloe vera* (AV) has garnered substantial attention in clinical research due to its extensive medicinal properties, including anti‐inflammatory, antimicrobial, wound‐healing, and immunomodulatory effects [11]. Its phytochemical constituents, such as anthraquinones, polysaccharides, and vitamins, contribute to its therapeutic potential, making it a promising candidate for managing various health conditions, including skin disorders, gastrointestinal ailments, and oral diseases [12]. The increasing evidence supporting AV’s efficacy underscores its potential as an adjunctive agent in clinical treatments, including periodontal therapy and other regenerative procedures [13]. The present study aims to assess the effects of local delivery of AV gel as an adjunct to scaling and RSD on clinical periodontal parameters (PI, gingival index (GI), BOP, probing pocket depth (PPD), and CAL), HbA1c, pro‐inflammatory cytokines (IL‐1β, IL‐6, and TNF‐α), and PGE2 in the treatment of type II diabetes patients with chronic periodontitis.

## 2. Materials and Methods

### 2.1. Setting and Design of the Study

The present study was carried out in the Diabetes Center (Shaheed Layla Qassim), BIO clinical laboratory, and Department of Periodontics, College of Dentistry/Hawler Medical University, Erbil, Kurdistan Region, Iraq.

A randomized controlled clinical study was conducted on eighty (80) periodontitis subjects’ stages 2 and 3 (moderate to severe) of both sexes (males and females), with an age range of 30–50 years. Forty patients were recruited with type II‐ partially controlled and uncontrolled diabetes (HbA1c >7%) and chronic periodontitis, and 40 systemically healthy participants with stages 2 and 3 periodontitis. The participants of this randomized controlled clinical study were randomly allocated into study groups using a sealed opaque envelope method to ensure unbiased group assignment.

The criteria applied for the diagnosis of diabetes were selected according to the American Diabetic Association criteria (HbA1c >7%), 2013. The sample were divided into four groups according to their glycemic status and type of treatment received. The study participants with type II diabetes and chronic periodontitis were randomly assigned (using a random number table) to two groups: Group I receiving AV gel as an adjunct treatment and Group II receiving chlorhexidine (CHX) as an adjunct treatment. Similarly, systemically healthy participants with chronic periodontitis were also assigned into two groups: Group III receiving AV gel as an adjunct treatment and Group IV receiving CHX as an adjunct treatment. The interventions assigned for the individual groups are as follows.

Group I. (Diabetic and periodontitis + *Aloe vera* Group—D.ALV) included 20 diabetic patients who received SRP + AV gel (98%).

Group II. (Diabetic and periodontitis + CHX Group—D. CHX). Included 20 diabetic patients who received SRP + 0.2% CHX gel.

Group III. (Periodontitis + AV Group—CP.ALV). Systemically healthy periodontitis group. Included 20 patients who received SRP + AV gel (98%).

Group IV. (Periodontitis + CHX Group, CP.CHX). Included 20 systemically healthy periodontitis patients who received SRP + 0.2% CHX gel.

### 2.2. Inclusion and Exclusion Criteria

Inclusion criteria for participants with chronic periodontitis included a PPD ≥5 mm and clinical attachment loss (CAL) ≥3 mm in at least 40% of teeth, and at least 20 remaining teeth. Diabetic participants (Groups I and II) were included if they had type II DM only, with no other systemic diseases, and had been on oral medications and dietary changes for type II diabetes for 1–5 years. Exclusion criteria encompassed allergies to study materials (CHX or AV), alcohol or tobacco use, recent (within 3 months) vitamin/mineral supplement use or antibiotic use, and recent (within 6 months) periodontal therapy.

### 2.3. Ethical Approval

This study was conducted in accordance with the ethical principles outlined in the Declaration of Helsinki. The protocol was reviewed and approved by the Ethics Committee of the College of Dentistry, Hawler Medical University (Ref. Number 11022, dated January 18, 2021). Written informed consent was obtained from all participants after they were provided with detailed information regarding the purpose and procedures of the study. Prior to group allocation, fasting blood sugar (FBS) and glycosylated hemoglobin (HbA1c) levels were evaluated for each subject.

### 2.4. AV Gel Preparation and CHX Gel Composition

AV gel (Barbadensis millar) was prepared in the College of Pharmacy, Hawler Medical University, Kurdistan Region, Erbil, Iraq, according to the procedure prescribed and outlined by Velam et al. [14]. The central parenchymatous pulp of the AV leaves was removed, and the pulp was washed many times using a 0.1 N sodium hydroxide (NaOH) solution and water before being blended to make a juice. First, a cotton bed was used to prefilter the juice and remove any leftover peel particles. The juice was then subjected to numerous vacuum filtrations until a clear liquid was obtained. The 1% w/w carbopol 934 was completely dissolved, leaving no lumps of carbopol behind. As the carbopol was dispersed, 0.5% w/w of methylparaben was added. Subsequently, a 0.5 N NaOH solution was added drop by drop until a gel took shape. To stop photooxidation, the gel was then weighed and put back in a sterilized dark container. While 0.2% CHX gel (Cervitec Gel, Ivoclar Vivadent, Liechtenstein) was utilized for CHX. CHX gel’s ingredients are 900 parts per million F, 0.2% CHX digluconate, aqua, hydroxyethyl cellulose, Laureth‐23, sodium fluoride, fragrance, CHX digluconate, and sodium saccharin [15].

### 2.5. Local Gel Delivery

#### 2.5.1. Local Gel Administration and Patient Instructions

Prepared ALV and CHX gels were locally administered following SRP into periodontal pockets at test and control sites, respectively, using a disposable syringe and a blunt cannula. Gel application occurred at baseline and 1 week later. Following each application, a periodontal dressing (Coe‐Pak) was placed over the treated sites for 7 days to retain the gel. Patients were instructed to avoid brushing or using interdental aids near the treated areas, refrain from chewing hard or sticky food, and avoid other chemical adjunctive hygiene agents during the dressing period. On Day 7, the dressing was removed, and the second gel application was performed, followed by another 7‐day period with Coe‐Pak and the same instructions. Throughout the 3‐month study duration, all participants were instructed to use the same toothpaste (Signal Family Protection toothpaste). Patients were recalled on Day 90 for final clinical periodontal parameter assessments and blood sample collection.

#### 2.5.2. Clinical and Biochemical Assessments

A single examiner assessed clinical periodontal parameters (PI, GI, BOP, PPD, and CAL) at baseline and 3 months. Measurements were taken at six sites per tooth (excluding third molars) using appropriate probes, and indices were scored according to established methods (Silness and Löe, 1964; Löe and Silness, 1963). Following baseline assessment and before intervention, all participants received SRP treatment; and instructions on the modified Bass brushing technique, with a directive to avoid mouth rinses.

Fasting venous blood samples were collected at baseline and 3 months. Serum was obtained by centrifugation and used for immediate FBS and HbA1c measurement, with aliquots stored at −20°C for subsequent analysis of PGE2, IL‐1β, TNF‐α, and IL‐6 levels via ELISA (Elabscience)

### 2.6. Statistical Analysis

Data were analyzed using SPSS, Statistical analysis was performed using IBM SPSS Statistics for Windows, Version 22.0 (IBM Corp., Armonk, NY, USA. Descriptive statistics included numbers, mean, and standard deviation. Normality was assessed with Shapiro–Wilk and Kolmogorov–Smirnov tests. Due to nonnormal data distribution, nonparametric tests were used. Within‐group comparisons (baseline vs. 3 months) were performed using the Wilcoxon signed‐rank test. Among‐group comparisons utilized the Kruskal–Wallis H test, with post hoc pairwise comparisons conducted using the Mann–Whitney *U* test. Statistical significance was set at *p* < 0.05.

## 3. Results

### 3.1. Male and Female Demographic Profile

Table 1 shows that the average age of the male and female participants was 45.38 ± 9.531 years. The women participants had an average age of 47.62 ± 11.24 years, while the men had an average age of 44.58 ± 8.81 years.

**Table 1:** The mean ages and standard deviation of male and female.

Sex	Number	Mean	SD	*T*‐Test	*p*‐Value
Male	59	44.58	8.811	−1.261	0.211
Female	21	47.62	11.249		
Total	80	45.38	9.531		

*Note: p*, probability ≤0.05 means statistically significant.

Abbreviations: N, number; SD, standard deviation.

After 3 months of applying AV and CHX gels to diabetic patients, Table 2 demonstrates a significant (*p* < 0.01) decrease in the mean levels of FBS and HbA1c in the diabetic AV (D.AV) and diabetic CHX (D.CHX) groups, respectively. The D.AV group showed the most mean differences (improvement) in FBS (47.445 mg/dL), while the D.CHX group showed the mean differences (5.600 mg/dL). In periodontitis patient groups (III and IV), both FBS and HbA1c were within the normal mean level of (91.6 ± 7.272 mg/dL) and (4.84 ± 0.462%) in CP.AV group and (97.4 ± 5.009 mg/dL) and (4.76 ± 0.335%) in CP.CHX group, respectively, with significant reduction after therapy, and the mean differences of FBS and HgA1c were higher in CP.CHX group, followed by CP.AV group. After 3 months, all four research groups showed nearly significant (*p* < 0.05) reductions in the periodontal parameters, within the D.AV group which, the GI showed nonsignificant (*p* > 0.05) results. Also, nonsignificant reductions in PI and BOP were observed in the D.CHX group.

**Table 2:** The mean serum level of fast blood sugar test and hemoglobin A1c at baseline (before therapy) and after therapy in both diabetes and periodontitis patients’ groups.

Groups	Blood sugar test	Mean ± SD before	Mean ± SD after	Mean difference	*Z*‐test (Wilcoxon signed ranks test)	*p*‐Value
Group I–D.AV	FBS (mg/dL)	257.83 ± 65.66	210.39 ± 49.321	**47.445**	−3.597	**<0.01**
	HbA1c (%)	8.34 ± 1.528	7.91 ± 1.516	0.439	−2.685	**<0.01**

Group II–D.CHX	FBS (mg/dL)	179.2 ± 39.965	173.6 ± 42.201	5.600	−3.540	**<0.01**
	HbA1c (%)	7.74 ± 1.5	6.94 ± 0.802	0.800	−2.979	**<0.01**

Group III–CP.AV	FBS (mg/dL)	91.6 ± 7.272	90 ± 5.62	1.600	−2.585	<0.05
	HbA1c (%)	4.84 ± 0.462	4.8 ± 0.445	0.040	−2.828	**<0.01**

Group IV–CP.CHX	FBS (mg/dL)	97.4 ± 5.009	89.8 ± 9.523	**7.600**	−3.937	**<0.01**
	HbA1c (%)	4.76 ± 0.335	4.54 ± 0.353	**0.220**	−3.982	**<0.01**

*Note: p*, probability ≤0.05 means statistically significant. Those values of mean differences in fasting blood suger and haemoglobin have been marked as bold which shows statistically significant difference (as shown by the *p*‐value, also marked as bold).

Abbreviations: CP.AV, chronic periodontitis *Aloe vera*; CP.CHX, chronic periodontitis chlorhexidine; D.AV, diabetes *Aloe vera*; D.CHX, diabetes chlorhexidine; FBS, fast blood sugar; HbA1c, hemoglobin A1c.

After 3 months, the D.AV group had greater mean differences in PI, PPD, CAl, and BOP than the D.CHX group (Table 3). Additionally, compared to the CP.CHX group, the Table displays larger mean differences for PPD, CAL, PPD, and BOP in the CP.AV group.

**Table 3:** The mean age’s ± standard deviation and the mean differences of clinical periodontal parameters before and after therapy in diabetes and periodontitis groups.

Groups	Index	Before mean ± SD	After mean ± SD	Mean difference	*Z*‐test (Wilcoxon signed ranks test)	*p*‐Value
Group I D.AV	PI	2.3 ± 0.186	2.05 ± 0.208	**0.250**	−3.947	**<0.01**
	GI	2.13 ± 0.262	2.03 ± 0.29	**0.100**	−1.475	0.14
	PPD (mm)	5.42 ± 0.437	4.33 ± 0.96	**1.083**	−3.976	**<0.01**
	CAL (mm)	4.75 ± 1.249	3.42 ± 1.206	**1.334**	−3.976	**<0.01**
	BOP (%)	76.22 ± 9.305	47.72 ± 15.303	**28.500**	−3.709	**<0.01**

Group II D.CHX	PI	2.12 ± 0.407	2.02 ± 0.582	0.100	−1.155	0.248
	GI	2.36 ± 0.264	2.04 ± 0.264	0.320	−3.959	**<0.01**
	PPD (mm)	5.8 ± 0.768	5.1 ± 1.536	0.700	−2.417	**<0.05**
	CAL (mm)	4.6 ± 0.821	4.12 ± 1.413	0.480	−2.189	**<0.05**
	BOP (%)	76 ± 23.93	72 ± 15.079	4.000	−1.176	0.24

Group III CP.AV	PI	1.68 ± 0.101	1.28 ± 0.164	0.400	−3.982	**<0.01**
	GI	1.56 ± 0.413	1.14 ± 0.448	0.420	−3.959	**<0.01**
	PPD (mm)	5.3 ± 0.41	4.5 ± 0.649	0.800	−4.053	**<0.01**
	CAL (mm)	4.6 ± 0.821	4.12 ± 1.413	0.480	−2.189	**<0.05**
	BOP (%)	69.9 ± 15.304	34.65 ± 11.708	35.25	−3.925	**<0.01**

Group IV CP.CHX	PI	1.64 ± 0.239	1.08 ± 0.209	0.220	−3.959	**<0.01**
	GI	1.8 ± 0.554	1.58 ± 0.375	0.22	−2.585	**<0.05**
	PPD (mm)	4.2 ± 0.41	3.3 ± 0.41	0.900	−4.179	**<0.01**
	CAL (mm)	3.4 ± 1.046	2.4 ± 1.046	1.000	−4.472	**<0.01**
	BOP (%)	72 ± 24.623	58 ± 19.894	0.560	−3.300	**<0.01**

*Note: p*, probability ≤0.05 means statistically significant. Those values of mean differences in clinical periodontal parameters have been marked as bold which shows statistically significant difference (as shown by the *p*‐value, also marked as bold).

Abbreviations: BOP, bleeding on probing; CAL, clinical attachment loss; CP.AV, chronic periodontitis *Aloe vera*; CP.CHX, chronic periodontitis chlorhexidine; D.AV, diabetes *Aloe vera*; D.CHX, diabetes chlorhexidine; GI, gingival index; PI, plaque index; PPD, probing pocket depth.

Regarding the immunological parameters (Table 4), diabetes patients’ mean serum levels of PGE2, IL‐1β, TNF‐α, and IL‐6 were significantly lower after 3 months of therapy in the D.AV group (Group I) compared to the base line prior to therapy (*p* < 0.05). However, following CHX gel local application in the D.CHX group (Group II), the mean serum levels of IL‐1β, TNF‐α, and IL‐6 were also decreased but showed nonsignificant differences with the base line (*p* > 0.05). PGE2 and pro‐inflammatory cytokines showed greater mean changes (improvements) in the D.AV group compared to the D.CHX group among diabetes patients.

**Table 4:** The mean age’s ± standard deviation, and the mean differences of PGE2 and pro‐inflammatory cytokines before and after therapy in diabetes and periodontitis patients groups.

Groups	Parameters	Before mean ± SD	After mean ± SD	Mean difference	*Z*‐test (Wilcoxon signed ranks test)	*p*
Diabetes						
Group I D.AV	PGE2 (pg/mL)	45.73 ± 12.192	33.23 ± 7.962	**12.501**	−3.811	**<0.01**
	IL1‐β (pg/mL)	210.16 ± 19.167	191.29 ± 14.562	**18.867**	−3.589	**<0.01**
	TNF‐α (pg/mL)	30.89 ± 9.956	20.14 ± 2.357	**10.756**	−3.485	**<0.01**
	IL‐6 (pg/mL)	2.6 ± 1.781	2.08 ± 1.357	**0.525**	−2.019	**<0.05**
Group II D.CHX	PGE2 (pg/mL)	38.25 ± 14.1	34.73 ± 19.892	3.517	−2.133	**<0.05**
	IL1‐β (pg/mL)	212.62 ± 27.277	206.67 ± 41.164	5.956	−1.853	0.064
	TNF‐α (pg/mL)	24.2 ± 4.125	22.73 ± 2.284	1.467	−1.532	0.126
	IL‐6 (pg/mL)	1.27 ± 0.21	1.17 ± 0.22	0.156	−0.636	0.525

Periodontitis						
Group III CP.AV	PGE2 (pg/mL)	48.36 ± 18.089	35.58 ± 14.886	**12.780**	−2.962	**<0.01**
	IL1‐β (pg/mL)	227.52 ± 38.91	203.5 ± 40.498	**24.020**	−3.562	**<0.01**
	TNF‐α (pg/mL)	25.92 ± 4.734	20.94 ± 2.156	**4.980**	−3.937	**<0.01**
	IL‐6	1.02 ± 0.116	0.86 ± 0.041	0.156	−3.937	**<0.01**
Group IV CP. CHX	PGE2 (pg/mL)	35.14 ± 9.843	27.68 ± 4.08	12.340	−1.987	**<0.05**
	IL1‐β	205.64 ± 24.589	193.3 ± 16.61	3.520	−3.937	**<0.01**
	TNF‐α (pg/mL)	25.26 ± 6.644	21.74 ± 1.818	0.110	−2.186	**<0.05**
	IL‐6 (pg/mL)	1.09 ± 0.107	0.98 ± 0.068	1.600	−2.962	**<0.01**

*Note: p*, probability ≤0.05 means statistically significant. Those values of mean differences in PGE2 and pro‐inflammatory cytokines have been marked as bold which shows statistically significant difference (as shown by the *p*‐value, also marked as bold).

Abbreviations: BOP, bleeding on probing; CAL, clinical attachment loss; CP.AV, chronic periodontitis *Aloe vera*; CP.CHX, chronic periodontitis chlorhexidine; D.AV, diabetes *Aloe vera*; D.CHX, diabetes chlorehexidine; FBS, fast blood sugar; GI, gingival index; HbA1c, hemoglobin A1c; IL‐1 β, interlukin I beta; IL‐6, interlukin 6; PGE2, prostaglandin E2; PI, plaque index; PPD, probing pocket depth; TNF‐α, tumor necrosis factor alpha.

The mean serum levels of PGE2, IL‐1β, TNF‐α, and IL‐6 in periodontitis patient groups were significantly reduced after therapy in both CP.AV and CP.CHX groups (*p* < 0.05). However, CP.AV group (III group) had higher mean differences in PGE2 and pro‐inflammatory cytokines levels than CP.CHX group (IV group), with the exception of CP.CHX group’s greater mean difference in IL‐6 levels in periodontitis patients.

Out of the four groups, the group with the largest mean improvements in PGE2 was CP.AV (12.780), followed by D.AV (12.501), CP.CHX (12.340), and D.CHX (3.517). The CP.AV group had the largest mean improvement (24.020) for IL1‐β, followed by the D.AV (18.867), D.CHX (5.956), and CP.CHX (3.520) groups. As indicated in Table 4, the groups with the highest mean improvement in TNF‐α were D.AV (10.756), CP.AV (4.980), D.CHX (1.467), and CP.CHX (0.110). In terms of IL‐6, the groups with the highest mean improvement were CP.CHX (1.600), D.AV (0.525), CP.AV (0.156), and D.CHX (0.156).

For the comparison among four groups and between each of the two groups before and after therapy Kruskal –Wallis test was performed. Table 5 shows that no significant differences were detected among four groups regarding BOP, TNF‐α, and IL‐1β before therapy (*p* > 0.05). In diabetes patients groups, no significant difference was detected between D.AV and D.CHX groups regarding HbA1c, PI, BOP, PI, PPD, CAL, PGE2, and IL‐1β groups (*p* > 0.05) except the significant differences between D.AV and D.CHX regarding FBS, GI and TNF‐α (*p* < 0.05). In periodontitis patient groups, no significant differences were detected between CP.AV and CP.CHX regarding HbA1c, clinical, and immunological parameters (*p* > 0.05) except the significant differences between both groups regarding FBS, PPD, and CAL, also no significant differences were detected between D.AV and CP.CHX regarding GI, BOP, and IL‐1β (*p* > 0.05). After therapy, Table 5 shows no significant differences were detected among the four groups regarding PGE2 and IL‐1β only (*p* > 0.05), and for the comparison between the two groups. no significant differences were detected: between D.AV and D.CHX groups regarding HbA1c, PI, GI, BOP, PPD, CAL, IL‐1β, and PGE2, between D.AV and P.CHX regarding IL1β and between P.AV and P.CHX regarding FBS, PGE2, IL‐1β, and TNF‐α (*p* > 0.05). Therefore, AV can be administrated locally instead of CHX for the healing of clinical and immunological parameters in type II diabetes and CP patients.

**Table 5:** A nonparametric test for the comparison between the mean values of each two groups using the Mann–Whitney *U* test and among four groups using the Kruskal–Wallis H test before and after therapy.

Indices	Group vs. group^a^					*p* ^∗^
	D.AV vs. D.CHX	D.AV vs. CP.AV	D.AV vs. CP.CHX	D.CHX vs. CP.AV	CP.AV vs. CP.CHX	
Before therapy						
FBS	HS	HS	HS	HS	S	0.001
HbA1c	NS	HS	HS	HS	NS	0.001
PI	NS	HS	HS	HS	NS	0.001
GI	HS	HS	NS	HS	NS	0.001
BOP	NS	NS	NS	NS	NS	0.720
PPD	NS	NS	HS	S	HS	0.001
CAL	NS	NS	HS	NS	HS	0.001
PGE2	NS	NS	HS	NS	NS	0.023
IL‐1β	NS	NS	NS	NS	NS	0.225
TNF‐α	S	NS	S	NS	NS	0.090
IL‐6	HS	HS	HS	HS	NS	0.001
After therapy						
FBS	S	HS	HS	HS	NS	0.001
Hb A1c	NS	HS	HS	HS	S	0.001
PI	NS	HS	HS	HS	HS	0.001
GI	NS	HS	HS	HS	HS	0.001
BOP	HS	HS	S	HS	HS	0.001
PPD	NS	NS	HS	NS	HS	0.001
CAL	NS	NS	S	NS	HS	0.001
PGE2	NS	NS	HS	NS	NS	0.266
IL‐1β	NS	NS	NS	NS	NS	0.686
TNF‐α	HS	NS	HS	S	NS	0.006
IL‐6	S	HS	HS	HS	HS	0.001

*Note:* *p* ≤ 0.05 means statistically significant.

Abbreviations: BOP, bleeding on probing; CAL, clinical attachment loss; FBS, fast blood sugar; GI, gingival index; HbA1c, hemoglobin A1c; HS, high significant; IL‐1β, interleukin I beta; IL‐6, interlukin 6; NS, nonsignificant; PGE2, prostaglandin E2; PI, plaque index; PPD, probing pocket depth; S, significant; TNF‐α, tumor necrosis factor alpha.

^a^Mann–Whitney *U* test.

^∗^Kruskal–Wallis test.

## 4. Discussion

Periodontitis is a plaque‐induced inflammatory and infectious disease linked to noncommunicable diseases such as cardiovascular diseases [16] and diabetes, with a specific bidirectional relationship noted between type II diabetes and periodontal disease. Glycemic management independently influences periodontal inflammation [17]. The study’s purpose was to assess the effectiveness of local ALV gel with SRP for treating chronic periodontitis in patients with type II diabetes.

### 4.1. AV Gel and the Level of FBS and HbA1c

Significant reductions in FBS and HbA1c were observed in diabetic patients using ALV gel. Potential explanations for AV’s antidiabetic effects include its strong antioxidant properties. AV suppresses free radicals and improves cellular thiol status [18]. This antioxidant potential could contribute to an antidiabetic effect, considering the role of oxidative stress in pancreatic β‐cell destruction [19] and diabetes pathogenesis. A second possible explanation is AV’s anti‐inflammatory properties [20]. Diabetes is seen as an inflammatory disease where inflammation contributes to progression, and TNF‐α decreases peripheral insulin sensitivity [21]. AV’s anti‐inflammatory properties are attributed to constituents like emodin and mannose‐6‐phosphate [22]. Our findings align with Arora et al. [23] who reported significant FBS drops after oral AV. Studies suggest 2 months of AV gel extract may lower HbA1c and FBS by enhancing insulin resistance [24]. Dick et al. [25] also found oral AV significantly lowered HbA1c and FBS in subgroups. A narrative review conducted by Cosola et al. [26] highlighted the importance of glycemic control and other risk factors in improving the overall outcomes in diabetic patients. Use of AV as an adjunct in such patients may contribute positively in this regard.

Following local application of CHX gel in diabetic patients, the current study observed significant improvements in FBS and HbA1c levels, although the difference in HbA1c improvement between the CHX and AV groups was not statistically significant. The antibacterial effects of CHX, which reduces Gram‐negative bacteria and plaque, likely contributed to decreased gingival inflammation and subsequently improved insulin resistance. These findings are consistent with previous research indicating that antimicrobial effects of CHX can lead to better metabolic control over 3–6 months in diabetic patients [27]. Another study conducted by Marconcini et al. [28] evaluated the effects of nonsurgical periodontal treatment in type‐2 diabetic patients. The results of this study indicated that CHX mouth was reduced systemic oxidative potential and inflammation in such patients and facilitated glycemic control [28].

### 4.2. Periodontal Parameters, Pro‐Inflammatory Cytokines, and PGE2

Application of AV gel in patients with periodontitis resulted in significant improvements in periodontal parameters. The CP.AV group showed the greatest reduction in plaque index (PI) and bleeding on probing (BOP), followed by other groups. For the GI, the D.AV group had the most improvement, while the CP.AV group showed the least. In terms of pocket depth and CAL, the D.AV group also demonstrated the greatest improvements. AV’s beneficial effects are likely due to its antibacterial, anti‐inflammatory, and wound‐healing properties, attributed to its rich content of vitamins, anthraquinones, glycoproteins, minerals, and amino acids.

The current study aligns with previous research showing that periodontitis patients treated with subgingival AV gel for one or 3 months experienced significant improvements in GI, PPD, and PI, attributed to the gel’s anti‐inflammatory, antibacterial, and wound‐healing properties [29]. The reduction in GI may also be linked to compounds like lupeol, an antiseptic analgesic, and sterols, which possess anti‐inflammatory effects. Vazquez et al. [30] reported that AV inhibits polymorphonuclear leukocyte (PMNL) migration and decreases edema and neutrophil counts.

Pradeep et al. [31] found that applying topical AV gel alongside SRP in patients with type‐2 diabetes and periodontal disease resulted in significantly reduced plaque and probing depths compared to a placebo. Similarly, Vangipuram et al. [32] reported that AV mouthwash was as effective as CHX in improving periodontal health, notably enhancing clinical attachment levels over 3 months.

Our study indicated that subgingival application of CHX gel in periodontal patients effectively improved clinical parameters such as PI, GI, BOP, pocket probing depth, and clinical attachment level. This is likely due to its ability to maintain effective CHX concentrations in the periodontal pockets, enhancing the benefits of SRP [33]. The positive effects of CHX gel can last up to 90 days [34]. Additionally, diabetic patients in the D.CHX group also showed improvements in gingival health, pocket depth, and attachment levels following treatment.

### 4.3. Effect of AV Gel on the Inflammatory Biomarkers

The study found that application of AV gel significantly reduced levels of inflammatory mediators such as PGE2 and IL‐1β in the CP.AV group, with greater improvements observed compared to the D.AV group. Similarly, TNF‐α levels decreased notably in the D.AV group following AV gel application, with the CP.AV group also showing significant improvement. These effects are likely due to AV’s anti‐inflammatory and antioxidant properties, which inhibit neutrophil migration and the production of pro‐inflammatory cytokines. AV enhances the body’s antioxidant capacity by decreasing lipid oxidation and boosting antioxidant enzyme activity [35]. Additionally, it suppresses inflammatory responses in immune cells by down regulating MMP‐9 and reducing cytokine production, such as IL‐1β and TNF‐α [36]. Its anti‐inflammatory effects also involve blocking the cyclooxygenase pathway, thereby lowering prostaglandin E2 (PGE2) levels, which helps reduce inflammation. The presence of compounds in AV that inhibit arachidonic acid oxidation further contributes to its anti‐inflammatory action.

A study revealed that the topical administration of AV gel in conjunction with SRP significantly improved clinical periodontal parameters (GI, PPD, and CAL) and significantly reduced IL‐1β and IL‐17 in the gingival crevicular fluid of patients with chronic periodontitis [37]. An additional investigation verified that Alo vera demonstrated anti‐inflammatory properties in vitro by impeding the generation of pro‐inflammatory cytokines (TNF‐α and IL‐6), mediators (NO, iNOS, and COX‐2) and by suppressing nuclear transcription factor kappa B (NF‐κB) and mitogen‐activated protein kinase (MAPK) signaling pathways [38].

The antibacterial and anti‐inflammatory properties of CHX may account for the present study’s findings that the pro‐inflammatory cytokines were also significantly reduced in the CP.CHX group following CHX gel application, and the inflammatory mediator PGE2 was also significantly reduced in the D.CHX and CP.CHX [39]. Over the whole course of the current investigation, no pain, hypersensitivity, or aberrant tissue reactions were noted.

A recent randomized controlled trial compared the efficacy of probiotic, AV, povidone‐iodine, and CHX mouthwashes in managing gingival inflammation, and demonstrated that all tested agents were effective, with probiotics and AV showing promising results as natural alternatives to conventional chemical mouthwashes [40]. These findings support our results and further highlight the potential role of adjunctive agents beyond CHX in improving gingival health.

Herbal formulations have been increasingly explored as alternative or adjunctive approaches in periodontal therapy, with several studies highlighting their anti‐inflammatory and antimicrobial [41] Herbal treatments used as an alternative in the treatment of periodontal diseases.

Our study has shown overall promising results with regards to the use of AV gel as an adjunct to SRP procedures. Furthermore, in the present study, adjunctive use of AV gel during nonsurgical periodontal therapy showed no local or systemic adverse effects throughout the follow‐up period. No allergic reactions, mucosal irritation, or sensitivity were reported. These findings align with recent clinical evidence supporting the safety of AV as a well‐tolerated herbal adjunct in periodontal treatment [42, 43].

However, there are some limitations which could have affected our outcomes. This was a single‐center study that included 20 patients per group. A multicenter trial would increase the overall sample size of the study and would also offer more representative sample for the particular city and this should be considered in the future. In this study, the investigator was not blinded and this could have induced bias in the results. Future studies should include double‐blinding to remove this bias and improve the overall quality of the study. Finally, a longer follow up of patients is required to eliminate any long‐term effects of the use of AV gel in the treatment of diabetic patients with chronic periodontitis.

## 5. Conclusions

According to the current findings, subgingival application of AV gel as an adjuvant to RSD showed a significant anti‐inflammatory effect and may help patients with type‐2 diabetes and chronic periodontitis by improving their periodontal disease. This is demonstrated by the gel’s significant positive effect on reducing hemoglobin A1c, fast blood sugar, clinical periodontal parameters, pro‐inflammatory cytokines (IL1β, TNF‐α, and IL‐6) and PGE2. AV gel shows potential as a safe and effective adjunct to RSD in diabetic patients with periodontitis. However, future studies with larger sample sizes and extended follow‐up are warranted to validate these findings.

## Author Contributions

Conceptualization, writing – review and editing: Chenar A. Mohammad and Faraedon M. Zardawi. Methodology: Chenar A. Mohammad and Hozan W. Aziz. Validation, visualization: Faraedon M. Zardawi. Formal Analysis: Aveen A. Jalal. Investigation: Hozan W. Aziz. Data Curation: Chenar A. Mohammad and Aveen A. Jalal. Writing – original draft, supervision, project administration: Chenar A. Mohammad.

## Funding

This research received no external funding.

## Ethics Statement

The study protocol was written according to the World Medical Association Declaration of Helsinki Ethical Principles for Medical Research Involving Human Subjects 2013. The study proposal was reviewed and approved by the Research Ethics Committee of the College of Dentistry, Hawler Medical University (Proposal Number 11022—dated January 18, 2021).

## Consent

The participants provided their written informed consent to participate in this study.

## Conflicts of Interest

The authors declare no conflicts of interest.

## Data Availability

All the raw data are available with the authors upon a reasonable demand.
